# Autoantibodies as prognostic markers in rheumatoid arthritis

**DOI:** 10.1016/j.jtauto.2025.100291

**Published:** 2025-05-03

**Authors:** Carl Turesson, Johan Rönnelid, Alf Kastbom

**Affiliations:** a Rheumatology, Department of Clinical Sciences, Malmö, Lund University, Malmö, Sweden; b Department of Rheumatology, Skåne University Hospital, Malmö, Sweden; c Department of Immunology, Genetics and Pathology, Uppsala University, Uppsala, Sweden; d Department of Clinical Immunology and Transfusion Medicine, Uppsala University Hospital, Uppsala, Sweden; e Department of Rheumatology, Linköping University Hospital, Linköping, Sweden; f Department of Biomedical and Clinical Sciences, Linköping University, Linköping, Sweden

**Keywords:** Rheumatoid arthritis, Rheumatoid factor, Anti-citrullinated peptide antibodies, Prognosis, Rapid radiographic progression, Extra-articular manifestations

## Abstract

**Background and purpose:**

Rheumatoid arthritis (RA) is a chronic inflammatory disorder characterized by progressively destructive polyarthritis. Key autoimmune features include the presence of autoantibodies. The purpose of this review is to discuss the diagnostic and prognostic properties of rheumatoid factor (RF) and anti-citrullinated protein/peptide antibodies (ACPA), based on current use in Sweden. Furthermore, we discuss their relation to disease outcomes and their importance for management of patients with RA.

**Principal findings:**

Based on current cut-offs, ACPA has a higher specificity for RA than RF, and testing for ACPA alone is recommended when investigating patients with clinically suspect RA. The diagnostic utility of RF may improve with adjusted reference range/upper limit of normal.

RF and ACPA both predict rapid radiographic progression, severe extra-articular manifestations and mortality, whereas other outcomes, such as osteoporosis, pain and disability are not as clearly related to seropositivity. RF/ACPA positive patients respond better to some targeted therapies, in particular rituximab, compared to seronegative RA patients. Recent studies indicate that treatment of ACPA-positive arthralgia with abatacept may delay and perhaps sometimes even prevent development of arthritis. Available evidence does not support an added value of repeated RF or ACPA testing.

**Conclusions:**

Testing for ACPA in patients with arthralgia or suspected early RA, and for ACPA and RF at RA diagnosis, provides useful diagnostic and prognostic information, which has implications for follow-up and treatment. Repeated testing for ACPA and RF is not recommended. Potential future developments include treatment of ACPA-positive individuals for prevention of arthritis.

## 1 Rheumatoid arthritis – a clinical syndrome with autoimmune features

Rheumatoid arthritis (RA) is considered a distinct disease entity in modern rheumatology. The basis for this is the recognition of a clinical syndrome of chronic polyarthritis with a progressive course. The very first description of RA is credited to a case series in the thesis presented by Augustin Jacob Landré-Beauvais in Paris in 1800 [1], although there may have been earlier case descriptions in the literature [2].

Subsequent work distinguished RA from infectious arthritis and crystal arthropathies, and later from various forms of spondyloarthritis (SpA), with prominent axial involvement and entesopathy. To this day, suspicion of RA is usually based on a history of gradually increasing peripheral joint pain together with a clinical examination indicating arthritis with a typical pattern of joint involvement. However, scientific progress on the underlying immunologic mechanisms has contributed immensely to diagnostic procedures and treatment of RA. Circulating autoantibodies have proven to be very useful for refining the definition of RA, and, in clinical practice, for early detection and for prognostics with implications for therapy.

The purpose of this review is to discuss the diagnostic and prognostic properties of the key autoantibodies associated with RA from our Swedish perspective, and to review their relation to disease outcomes and their importance for management of patients with RA.

## 2 Development of RF and ACPA assays

The initial assays for rheumatoid factor (RF) were based on the autoantibodies’ capacity to agglutinate red blood cells or latex-containing particles coated with IgG. Also nephelometry and turbidometry rely on agglutination. Although all these assays formally are isotype-nonspecific, they all mainly detect IgM RF. The development of isotype-specific enzyme immunoassays (EIA) made it possible to investigate individual RF isotypes separately, and many studies have confirmed that IgM RF has the highest diagnostic sensitivity for RA in Caucasian populations [3]. Within the Swedish External Quality Assessment program EQUALIS, most laboratories today utilize the same IgM-specific EIA (Phadia Elia, 15 laboratories), the remaining three laboratories use different automated turbidometry systems.

All assays for anti-citrulline protein/peptide antibodies (ACPA) rely on antigens, mostly proprietary peptides, bound to a solid phase, including on surfaces of beads used in addressable laser bead immunoassays (ALBIA). The first assays used citrullinated filaggrin or a peptide thereof made cyclic, forming the first cyclic citrullinated peptide denoted CCP1 [4,5]. These have today been replaced by proprietary (mixes of) cyclic peptides denoted CCP2 and CCP3. In Europe anti-CCP2 [6] assays provided by different companies dominates, whereas the anti-CCP3 [7] test is common in the United States. A German company has also developed an assay denoted anti-MCV based on mutated and citrullinated vimentin [8]. Although ACPA of different isotypes are produced, only IgG ACPA are determined in the absolute majority of assays. All Swedish clinical laboratories utilize IgG anti-CCP2 tests, albeit from different manufacturers.

Both RF and ACPA have WHO reference preparations but at present, only the first is in use. The RF standard was collected in 1963 and divided into three batches denoted W1066 [9], 64/002 and 64/003, all with the same unitage. Only 64/003 remains today. The absolute majority of RF assays have been standardized against this reagent, and the unitage consequently given in international units (IU)/mL. It is worth noting that the RF standard was prepared and characterized only with sheep cell agglutination, primarily measuring IgM RF activity, long before the first description of the ELISA assay at Stockholm University in 1971 [10]; the reagent also contains only small amounts of IgA RF and IgG RF. Thus 64/003 is not optimal for the determination of unitage isotype-specific assays except for IgM RF.

The first WHO international standard for ACPA became available in 2023 and is denoted 18/204. No commercial ACPA test has so far adopted the corresponding IU/mL unitage, instead, all ACPA assays use their own individual arbitrary unitages. Both the original investigation [11] and a later report [12] have shown that 18/204 works well with different anti-CCP2 assays but produce divergent results both for anti-CCP3 and anti-MCV.

Reference ranges are adopted differently for RF and for ACPA. The previous American College of Rheumatology RA classification criteria from 1987 included only RF in the serology domain, and stated clearly that the reference range or cut-point for a positive reaction should be defined so that <5 % of a healthy population became positive, corresponding to a diagnostic specificity of >95 % [13]. Consequently, most RF assays have a diagnostic specificity of around 95 %, and in fact quite even often lower than that. When ACPA were described around the turn of the century in Nijmegen and in Toulouse respectively, both research groups used higher diagnostic specificities around 98–99 % in relation to healthy controls [5,14], and this high-specificity approach has been adopted by assay manufacturers. The newer 2010 European Alliance of Associations for Rheumatology (EULAR)/ACR classification criteria for RA [15] include both RF and ACPA, but, in contrast to the former ACR criteria, they give no directions concerning how to define a positive response. By stating a reaction above the “upper limit of normal” (ULN) as a positive reaction they leave this decision at the discretion of the clinical laboratory providing the assay results. Due to these factors, RF assays usually still have a much lower diagnostic specificities as compared to ACPA assays.

Despite longstanding “standardization” of RF assays with a WHO reagent, individual commercial RF and ACPA assays differ considerably in their cutoffs, when compared to the same reference reagents. When compared to W1066, the serum levels of RF differ up to 15 times between commercial RF assays [16], while comparisons of different ACPA assays ACPA reference reagents showed >5-fold [17] and 2-fold [16] variations. According to the 2010 EULAR/ACR RA classification criteria 6 points (out of 10 possible) are needed for RA classification, and a patient is assigned 3 points or 50 % of what is needed for RA classification if either RF or ACPA levels are >3x above the ULN. If the corresponding levels are between the ULN and ≤3 times the ULN, 2 points are afforded, with no points for negative results. As the cutoffs between different RF and ACPA assays differ >3 times, the outcome of RA classification therefore depend heavily on what autoantibody assays are used [15,16].

Based on such experiences, the current view is that autoantibody analyses probably cannot be standardized like conventional clinical chemistry analyses, and that the goal instead should be to harmonize reporting and interpretation of autoantibody analyses [18]. One way to do this is via the introduction of test interval-specific likelihood ratios. Such ready-to-implement likelihood ratio data for common RF and ACPA assays were recently published [19].

## 3 Diagnostic use of RF and ACPA in Sweden

The positive predictive value (PPV) of a test depends on the test's diagnostic sensitivity and diagnostic specificity as well as on the frequency of patients with the target diagnosis in the investigated population. This implies that assays with low diagnostic specificity, like RF, will yield low PPV especially in populations with a small fraction of patients actually having RA, like in primary care settings. One US study reported a PPV for RF of 24 % from a university hospital setting [20]. A large retrospective population-based registry study from Denmark where 5 % of the investigated patients developed RA reported a PPV of 30 % for ACPA and as low as 12 % for IgM RF [21]. A PPV of 12 % can be translated that out of 8 RF positive patients investigated, less than one will develop RA.

In Sweden, ACPA was recommended to replace RF in primary healthcare when investigating cases of clinically suspect RA already 20 years ago [22]. This recommendation was implemented early and is still valid, as described in guidelines from the National Board of Health as well as in national textbooks in rheumatology and laboratory medicine. Due to the lower specificity of RF, this analysis is not recommended in cases with very early signs of suspect arthritis. If the patient on the contrary has persistent polyarthritis (high pretest probability of RA), or if the patient is already clinically diagnosed with RA, then simultaneous testing of ACPA and RF is recommended. This is due to the added prognostic value of RF (see section 5).

The recommendation to primarily use ACPA in primary care can be implemented in different ways. In Falu Hospital, Falun, Sweden where one of the authors (JR) has been responsible for autoimmune analyses the following algorithm has been developed together with the local rheumatology clinic, and been used for many years: ACPA/anti-CCP2 is the only analysis directly and easily requestable for primary care physicians via the digital analysis ordering system. Every sample that becomes anti-CCP2 positive is immediately also analysed for IgM RF, so that once the patient is referred to the rheumatologist both ACPA and RF status is known. There is a possibility for any doctor to order RF separately, but to prevent overuse, this is not a one-click maneuvre; this possibility is mostly utilized by the rheumatology specialists.

Together with the national external quality assessment organization EQUALIS, Swedish clinical immunologists and rheumatologists are currently discussing the possibility to adjust the reference range for the IgM RF assay most commonly used in Swedish clinical laboratories to a diagnostic specificity in line with anti-CCP2. This approach will make the RF likelihood ratios come closer to, but not equalise the likelihood ratios for ACPA [7,16,23].

Moving from diagnosis to classification, a very relevant discussion has started recently concerning the possibility to give RF and ACPA (and their combination) different weights in modified classification criteria [24,25]. Modifications of classification criteria that have been updated fairly recently [15] might be a challenge, and it is likely more feasible to just quietly change the reference range/ULN.

## 4 Controversy over “seronegative RA”

Most patients with a well-founded clinical suspicion of RA will test positive for RF and/or ACPA. In samples of patients diagnosed by rheumatologists, the proportion with “seropositive” RA is usually between 55 and 70 %. In populations eligible for clinical trials, where there is selection for disease severity by requirement of current active arthritis, this percentage tends to be higher. Some trials even include RF and/or ACPA as inclusion criteria in order to obtain a more homogeneous study population, e.g. for testing therapeutic agents with modes of action that are thought to be more useful for patients with evidence of autoantibody production.

Besides RF and ACPA, some additional antibodies have been implicated as diagnostic and/or prognostic biomarkers in RA. This includes those directed against carbamylated or homocitrulline-containing proteins (anti-CarP) [26], peptidyl arginine deiminase-4 (anti-PD-4) [27] or glucose-6-phosphate isomerase (anti-GPI) [28]. Additional examples include antibodies against type-II collagen (anti-CII) [29], heterogeneous nuclear ribonucleoprotein A2 (RA33) [30] or against products of lipid degradation, malondialdehyde (MDA) and malondialdehyde-acetaldehyde (MAA) [31]. Although these autoantibodies overlap only partially with ACPA and RF, and might contribute to redefining some conventionally seronegative cases, they have not come into widespread clinical use.

The concept of “seronegative RA” as a distinct disease entity has been challenged [32]. It has been argued that patients diagnosed with RA in the absence of RF and ACPA make up a heterogeneous population, with variable phenotypes, disease mechanisms and outcomes. For example, a group of rheumatologists in central Finland conducted a structured re-evaluation of patients diagnosed with seronegative RA in their area [33]. Based on a follow-up visit after 10 years, a majority of the patients had been assigned other diagnoses, e.g. polymyalgia rheumatica, psoriatic arthritis (PsA), osteoarthritis or SpA, and some had experienced a transient course of arthritis.

By contrast, in a retrospective cohort study from the Mayo Clinic in Rochester, Minnesota, most patients diagnosed with seronegative RA were still on treatment with unchanged diagnosis after a median follow-up of 4 years [34], with a recent preliminary report on extended data indicating a persistent change of diagnosis in only 15 % over 10 years in this RA-population [35]. The discrepancy may reflect differences in clinical practice across countries and centers, and also more frequent challenging of diagnoses with active re-evaluation including prospective follow-up visits, as compared to retrospective reviews of records from the managing rheumatologist.

Some support for regarding seropositive and seronegative RA as separate diseases is derived from studies of genetics and environmental risk factors. Whereas RF/ACPA-positive RA is strongly linked to genetic variants of *HLA-DRB1*, the picture is completely different for seronegative RA [36]. Furthermore, smoking has in many studies been demonstrated to be a risk factor for seropositive RA, but not for seronegative RA [37,38]. An interaction between smoking and *HLA-DRB1* has been described, specifically for ACPA positive RA [39], possibly explained by increased citrullination in the bronchial mucosa in smokers and preferential binding of citrullinated peptides to RA-associated *HLA-DRB1∗04* subtypes. On the other hand, hormone related factors may play a greater role for predisposition to seronegative RA [40,41].

## 5 Evidence for prognostic value of RF and ACPA

### 5.1 Progression of joint damage

It is well established that both ACPA and RF are strongly associated with progressive joint damage [42]. It has been demonstrated that these autoantibodies are more important markers of rapid radiographic progression (RRP) than clinical parameters. For example, in a study from southern Sweden, where patients with early RA (<12 months symptom duration) were followed according to a structured program, and RRP was defined as an increase of >5 points/year over 5 years in the modified Sharp-van der Heijde score, RF and ACPA were both associated with a markedly increased risk of RRP (Table 1) [43]. By contrast, swollen and tender joint counts at inclusion did not predict RRP (Table 1). Similarly, in two independent cohorts of recent-onset RA diagnosed 2006–2011, *i.e.* in the era of tight control and widespread access to biologics, baseline ACPA still predicted radiographic progression although disease activity over time was similar [44].

**Table 1:** Baseline predictors of rapid radiographic progression of joint damage^a^ over 5 years in patients with early rheumatoid arthritis.

	Odds ratio	95 % CI
Male sex	1.06	0.47–2.37
Age (per SD)	1.20	0.81–1.77
IgM-RF positive	**5.70**	**1.90–17.10**
Anti-CCP2 positive	**6.04**	**1.98–18.47**
Erosive disease at first X-ray	2.29	0.95–5.53
ESR (per SD)	**1.89**	**1.33–2.69**
CRP > median^b^	**2.89**	**1.31**–**6.39**
Swollen joint count (per SD)	1.26	0.87–1.84
Tender joint count (per SD)	0.85	0.55–1.32

CI: Confidence interval; SD: standard deviation - age 15 years; ESR 26 mm/h; swollen joint count 4.9; tender joint count 5.8.

a Change of >5 points per year in total Sharp-van der Heijde score.

b 9 mg/l.

In analysis of a sample of consecutive patients with newly diagnosed RA, RF, but not ACPA, was associated with presence of erosions at the very first X-ray (Table 2) [45]. This association was independent of symptom duration (Table 2). Taken together with observed associations between IgM-RF and systemic inflammation in ACPA-positive early RA [46], this suggests that RF related immune complexes drive inflammation and joint damage at the early stages of RA. There are also several studies reporting an association between IgA RF and erosions in RA [47].

**Table 2:** Relation between baseline characteristics and erosions at diagnosis in patients with rheumatoid arthritis – logistic regression.

Variable	Unadjusted	Multivariate^a^
	Odds ratio (95 % CI)	Odds ratio (95 % CI)
Male sex	0.94 (0.48–1.83)	NI
Age at inclusion, per SD	1.16 (0.83–1.62)	NI
Symptom duration, per SD	**1.71 (1.27–2.32)**	**1.73 (1.27–2.35)**
RF positive	2.10 (0.99–4.20)	**2.17 (1.01–4.67)**
ACPA positive	1.36 (0.67–2.76)	NI
DAS28-CRP, per SD	1.32 (0.89–1.95)	NI
CRP, by quartile (mg/l)		
<3.2	1 (ref)	NI
3.2–9.6	1.11 (0.42–2.94)	
9.61–26.5	1.86 (0.75–4.61)	
>26.5	1.70 (0.68–4.26)	
ESR, by quartile (mm/h)		
<14	1 (ref)	NI
14–28	1.51 (0.54–4.25)	
28–49	2.12 (0.78–5.72)	
>49	1.76 (0.63–4.87)	

NI: Not included.

a Forward stepwise selection. Adjusted for all the variables in the column.

### 5.2 Severe extra-articular manifestations

**Fig. 1:**
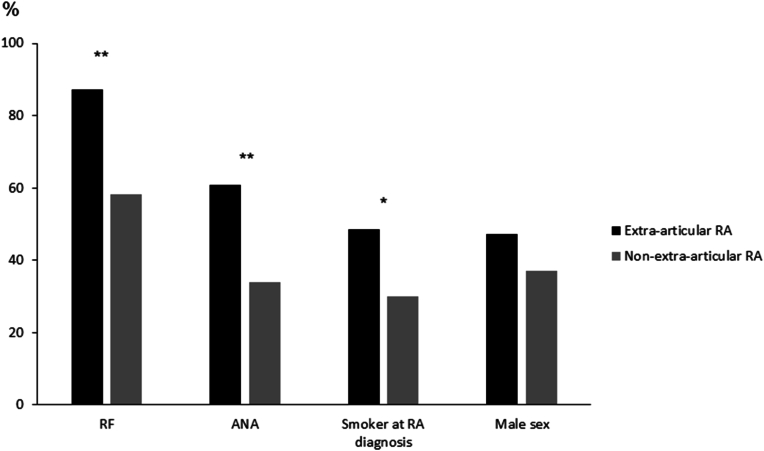
Autoantibodies and other baseline factors, by subsequent occurrence of severe extra-articular manifestations of RA. Data from a case-control study of patients with severe extra-articular RA (vasculitis, pericarditis, pleuritis, Felty's syndrome, scleritis, episcleritis, glomerulonephritis) and RA controls without such extra-articular manifestations, matched for duration of RA and centre. The sample was pooled from studies performed at the Mayo Clinic, Rochester, Minnesota, in Malmö and Lund, Sweden, and from the Swedish multicentre study BARFOT. ∗∗p < 0.001; ∗p = 0.001.

**Fig. 2:**
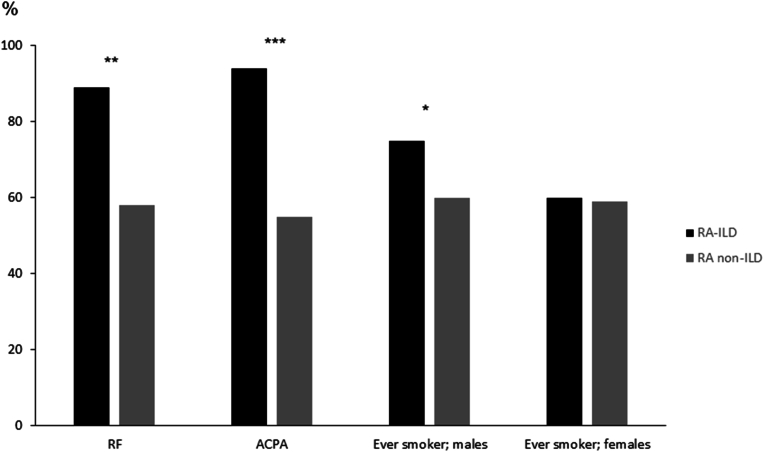
Baseline autoantibodies and history of ever smoking, by subsequent development of interstitial lung disease (ILD). Data from a large multi-centre study of patients with early RA in the United Kingdom, with structured long-term follow-up of the occurrence of ILD. ∗∗∗p = 0.006; ∗∗p = 0.01; ∗p = 0.02.

RA may sometimes be complicated by extra-articular organ involvement. This ranges from milder manifestations such as sicca syndrome and subcutaneous rheumatoid nodules to severe complications such as vasculitis, interstitial lung disease, pericarditis and scleritis. Such manifestations tend to cluster, i.e. occur together more frequently than expected based on chance, suggesting shared disease mechanisms [48]. This phenotype is clearly linked to seropositive RA. In a large multi-center study that pooled patients from several centers in Sweden and in the USA, 87 % of those with severe extra-articular RA (ExRA) were RF positive (Fig. 1) [49]. ExRA was also associated with the presence of anti-nuclear antibodies and with smoking, which as mentioned may contribute to disease mechanisms strictly related to seropositive RA (Fig. 1). In this analysis, many patients were diagnosed before the development of ACPA assays. However, a large multi-center study of early RA from the UK showed associations for both RF and ACPA with development of ILD, which was also associated with smoking in male patients [50] (Fig. 2).

Based on data from [49]

Based on data from [50]

Analyses of samples taken at the clinical presentation of severe ExRA manifestations have revealed higher levels of RF, but not ACPA, compared to RA controls without ExRA involvement [51]. Taken together, this suggests a role for RF-associated immune complexes in driving extra-articular manifestations.

Fortunately, the incidence of some ExRA manifestations, in particular vasculitis, appears to have decreased in recent years [52,53]. This has been attributed to improved pharmacologic treatment and better control of disease activity, and indicates that the presence of high levels of RF alone is not sufficient for development of severe ExRA organ involvement.

### 5.3 Mortality

Multimorbidity and mortality in patients with RA is clearly linked to disease severity, and there is also some evidence indicating that RF and high levels of ACPA may predict mortality [54]. In a large study based on two administrative claims databases in the United States, RF and ACPA positive RA patients had higher mortality rates compared to those lacking these antibodies, with significant differences in models adjusted for comorbidities, previous hospitalizations and current treatment [55]. There was also a reduced survival with higher compared to lower autoantibody levels. Interestingly, these associations were only demonstrated in patients never treated with biologic DMARDs, suggesting that they may be modified by active pharmacotherapy.

### 5.4 Other outcomes: osteoporosis, pain, hand function

**Fig. 3:**
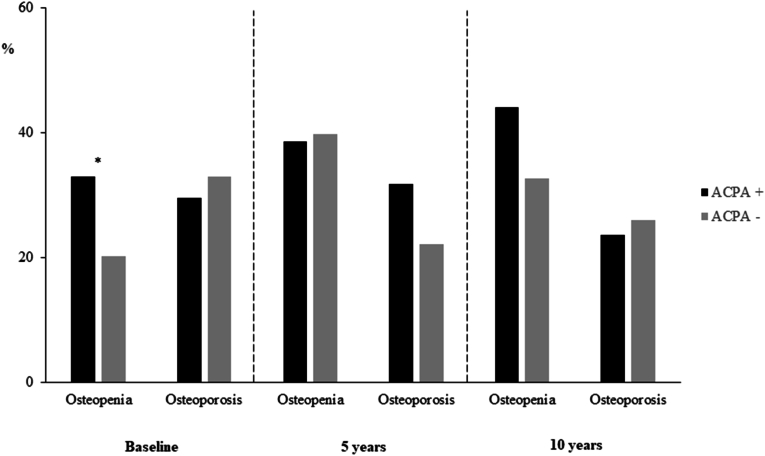
Prevalences of osteopenia and osteoporosis at follow-up visits in early RA, ACPA status. Osteopenia was defined as BMD T-score ≤ -1, but > -2,5. Osteporosis was defined as BMD T-score ≤ -2.5. Data from RA diagnosis and after 5 years and 10 years of follow-up in the Malmö early RA cohort. ∗p = 0.04.

As ACPA is clearly associated with joint erosions, i.e. juxta-articular bone loss, it has also been suggested that it may have an impact on general bone mineral density (BMD). In the study from southern Sweden where ACPA was strongly associated with RRP (Table 1), further analyses included repeated measures of BMD in the hip and the lumbar spine by dual X-ray absorptiometry [56]. There was an association between ACPA positivity and moderately reduced BMD (osteopenia) at diagnosis, but not at later time points (Fig. 3). Adjusted longitudinal analyses revealed a significantly lower BMD at inclusion, but not greater loss of BMD over time, in ACPA positive patients. Results were similar in identical analyses of a separate cohort from the Netherlands [57].

Based on data from [57]

**Fig. 4:**
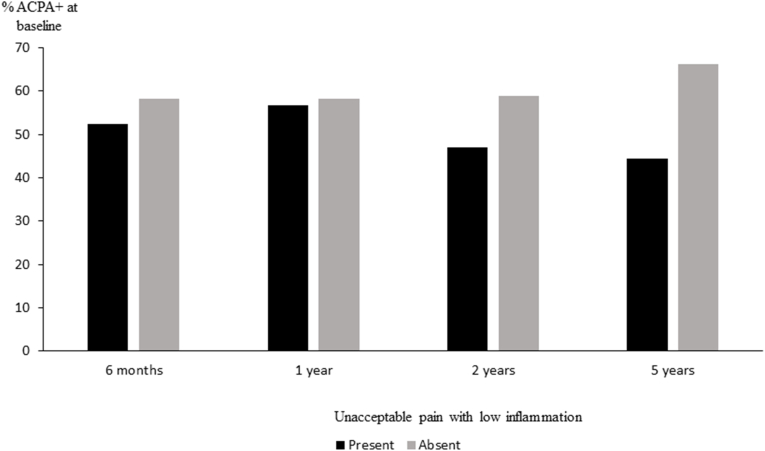
Proportions with positive ACPA at RA diagnosis by presence of unacceptable pain with low inflammation at follow-up. Unacceptable pain with low inflammation was defined as VAS pain>40 and CRP<10 mg/l at each follow-up visit up to 5 years. ACPA positive patients were less likely to experience unacceptable pain with low inflammation at 5 years (odds ratio 0.50; 95 % confidence interval 0.22–0.98).

Pain is an important symptom in RA, and may sometimes persist despite successful treatment of inflammation. Based on surveys of patient acceptable states, a score of <40 on a 0–100 visual analogue scale (VAS) has been defined as unacceptable pain [58]. In a structured follow-up of patients with early RA, patients experiencing unacceptable pain with low inflammation (CRP<10 mg/l) after 5 years were more likely to be ACPA-negative [59] (Fig. 4), suggesting that non-inflammatory long lasting pain may be a greater problem in this subset.

Based on data from [59]

Other important outcomes in RA include hand function and related impact on activities of daily living. Grip force is a standardized measure of hand function, which correlates strongly with patient reported disability [60]. In a study of patients with early RA, anti-CCP2 antibodies at baseline were not associated with reduced grip force (defined as <50 % of expected value, based on age and sex) after 5 years (odds ratio 0.99; 95 % CI 0.52–1.90) [54]. Other parameters, in particular current inflammation of the wrist or finger joints and pain, and possibly also factors related to coping and motivation that may affect patient performance at testing, may be more important for this type of outcome [61].

## 6 RF, ACPA and treatment response in RA

A pooled analysis of data from 16 national registers showed a major difference in the proportion attaining low disease activity for the B-cell depleting anti-CD20 antibody rituximab, with better outcome in seropositive patients [62] (Table 3). This is in agreement with previous results from both randomized controlled trials (RCTs) [63] and observational studies [64]. There were similar differences for the CTLA4-Ig fusion protein abatacept and for the interleukin-6 (IL-6) inhibitor tocilizumab, although with smaller magnitudes (Table 3). As rituximab depletes populations of active B-cells, abatacept blocks T-cell activation, indirectly influencing interaction between T-cells and antibody producing B-cells, and IL-6 is important for lymphocyte differentiation and activation, it is not surprising that these drugs should be somewhat more effective in patients with RA that are seropositive for ACPA or RF.

**Table 3:** Adjusted differences in proportions with LUNDEX corrected low disease activity∗ for patients with seropositive∗∗ vs. seronegative RA, for different biologic DMARDs. Pooled analysis from 16 European registers.

Drug/Class of drugs	Adjusted∗∗∗ difference – seropositive vs seronegative	95 % CI
Rituximab	11.6 %	9.3, 14.5
Abatacept	8.1 %	7.3, 8.9
Tocilizumab	5.1 %	3.9, 8.2
TNF inhibitor	−0.3 %	−0.8, 0.2

∗Clinical Disease Activity Index (CDAI) ≤ 10.

∗∗RF and/or ACPA positive.

∗∗ Adjusted for age, sex, smoking (yes/no), BMI for TNF inhibitors, abatacept and tocilizumab (but not for rituximab), for calendar year of treatment start, country, concomitant treatment with csDMARDs and glucocorticosteroids, number of previous bDMARDs and disease characteristics (baseline values for disease activity and disease duration) for all.

There was no such difference by seropositivity regarding low disease activity after treatment with TNF inhibitors (TNFi) (Table 3), as shown in other studies [[65], [66], [67]]**.** The efficacy of TNFi not only in treatment of RA, but also for seronegative conditions such as SpA, psoriasis, PsA and inflammatory bowel disease (IBD), is compatible with mechanisms of action not directly related to autoantibodies.

Data on drugs that block the intracellular Janus kinases (JAK), which were introduced more recently than the biologic DMARDs discussed above, are more limited. Results for tofacitinib from the phase III clinical trial program suggest that it may be slightly more effective in seropositive patients [68]. As JAK-inhibition has a wide variety of anti-inflammatory effects, and JAK-inhibitors have been shown to be effective also in the seronegative disorders SpA, PsA and IBD, a minor predictive effect of ACPA and RF would be expected in this context.

## 7 Treatment of ACPA positive arthralgia

Treatment of individuals with ACPA-positive arthralgia, but no clinical arthritis, has been evaluated in several double-blind RCTs. In the PRAIRI study, a single dose of rituximab (1000 mg intravenously) delayed onset of arthritis significantly [69]. However, after a follow-up of 4 years, the cumulative incidence of arthritis was similar to the placebo group (approximately 40 %). More recently, two trials of abatacept for such “at-risk” populations have been conducted [70,71]. In the first, presence of signs of synovitis, tenosynovitis or osteitis on magnetic resonance imaging of the dominant hand was part of the inclusion criteria [70]. In the second study, which may be considered to have included a “purer” arthralgia-only population, evidence of inflammation on imaging was not required, and systematic ultrasonography indicated that signs of synovitis were absent or minimal in the majority of included patients [71]. During the active treatment period (abatacept 125 mg subcutaneously once per week for 6 and 12 months, respectively), there were low rates of progression to clinical arthritis in the abatacept groups in both trials, and substantially higher rates in the placebo groups. During post-treatment observation, these differences were reduced, but remained significant after 1 year. Long-term follow-up studies (up to 8 years), and post-hoc evaluations of subsets with multiple autoantibodies that might have an even greater benefit from active treatment, are ongoing.

Studies of conventional synthetic DMARDs (hydroxychloroquine and methotrexate) for at-risk populations with less stringent inclusion criteria have not showed a significant benefit [72,73]. Taken together, this suggests that a well-defined immune phenotype and a therapy that targets key disease mechanisms are necessary for successful prevention of arthritis in individuals with ACPA-positive arthralgia. The benefits of preventing arthritis development need to be weighed against the potential risks of such treatment for individuals that have not been diagnosed with a chronic disorder, and treatment costs. Ironically, for abatacept, which has the strongest evidence base in this context, no biosimilar has been developed, contributing to persistently higher costs compared to other biologic DMARDs (e.g. TNF inhibitors and rituximab).

## 8 Value of repeated testing of RF and ACPA

There are several examples of rheumatic conditions where fluctuations in autoantibody levels are relevant to monitor due to their association with disease flares, e.g. antibodies to double-stranded DNA in systemic lupus erythematosus and anti-neutrophil cytoplasmic antibodies in granulomatosis with polyangiitis. Possibly due to extrapolation of this knowledge, re-testing is also frequently performed concerning RF and ACPA in patients with arthralgia or RA, although the scientific basis for doing so is much less clear. Nevertheless, retrospective biobank studies on asymptomatic pre-RA individuals showed that RF and ACPA levels positivity increase as time remaining to symptom onset and RA diagnosis decreases [[74], [75], [76]], indicating that it may be relevant to monitor autoantibody levels in the at-risk phase. Such reasoning was further substantiated by findings in ACPA-positive subjects with musculoskeletal symptoms, where baseline levels of both ACPA and RF levels were prognostic for progression to arthritis [77,78]. However, in prospective studies with longitudinal sampling in symptomatic subjects, no indications emerge that ACPA or RF levels change in any prognostically meaningful way, regardless of whether progression to arthritis occurs or not [[79], [80], [81]]. These rather surprising findings imply that, in a context where the patient sought health care due to arthralgia but does not present signs of arthritis, repeated RF and ACPA analyses should be avoided.

In the context of recent-onset arthritis, the value of repeated autoantibody testing was investigated in a large prospective French cohort of patients with ≥2 swollen joints and symptom duration <6 months where 80 % fulfilled 2010 ACR/EULAR criteria for RA. Here, it was clearly shown that ACPA and RF levels and status remained stable during 2 years of follow-up [82]. Similar findings were made in a Norwegian early arthritis cohort including patients with ≥1 swollen joint and symptom duration ≤16 weeks [83]. In contrast, a Canadian early arthritis cohort found that over 2 years, changes were not uncommon for ACPA (8 % turned negative and 11 % turned positive) and RF (28 % turned negative and 18 % turned positive). However, prognostic associations with such changes were generally absent [84].

In manifest RA, levels of both ACPA [[85], [86], [87]] and, seemingly to a greater extent, RF [85,87] decrease following DMARD treatment. There is some evidence that the degree of reduction in ACPA and RF associates with therapeutic response [85,86], suggesting a potential use of repeated testing to evaluate treatment outcomes. However, the observed associations between autoantibody reductions and treatment outcomes have been moderate [85,86], and inconsistent [[86], [87], [88]]. Taken together, despite the obvious prognostic value of baseline testing, we see no added value of repeating ACPA and RF analyses in neither symptomatic at-risk, recent-onset arthritis, nor early RA settings.

## 9 Conclusions

We suggest to only use ACPA when investigating cases of clinically suspect RA in primary care. The diagnostic utility of IgM-RF may benefit from adjusting the reference range to increase specificity. Presence of IgM-RF and ACPA at diagnosis predicts rapid progression of joint damage, severe extra-articular manifestations and premature mortality, but to a lesser degree other outcomes such as generalized bone loss, patient reported pain and disability. Targeted therapies that specifically affect lymphocyte functions may be more beneficial for RF/ACPA positive patients compared to those with seronegative RA.

Testing for RF and ACPA at RA diagnosis provides useful diagnostic and prognostic information, but repeated testing has not shown added value in patients with arthralgia or manifest RA.

## CRediT authorship contribution statement

**Carl Turesson:** Writing – review & editing, Writing – original draft, Visualization, Investigation, Conceptualization. **Johan Rönnelid:** Writing – review & editing, Investigation, Conceptualization. **Alf Kastbom:** Writing – review & editing, Investigation, Conceptualization.

## Funding

This research did not receive any specific grant from funding agencies in the public, commercial, or not-for-profit sectors.

## Declaration of competing interest

The authors declare the following financial interests/personal relationships which may be considered as potential competing interests:Carl Turesson reports a relationship with 10.13039/100006483AbbVie Inc that includes: consulting or advisory and speaking and lecture fees. Carl Turesson reports a relationship with Nordic Drugs AB that includes: consulting or advisory and speaking and lecture fees. Carl Turesson reports a relationship with 10.13039/100004319Pfizer Inc that includes: speaking and lecture fees. Alf Kastbom reports a relationship with 10.13039/100006483AbbVie Inc that includes: speaking and lecture fees. Johan Rönnelid reports a relationship with Thermo Fisher Scientific that includes: consulting or advisory. Johan Rönnelid reports a relationship with 10.13039/100011033Thermo Fisher Scientific that includes: speaking and lecture fees. Johan Rönnelid reports a relationship with Inova that includes: consulting or advisory. Johan Rönnelid reports a relationship with Boehringer Ingelheim Ltd that includes: speaking and lecture fees.

## Data Availability

No data was used for the research described in the article.
